# Monocytes Undergo Functional Reprogramming to Generate Immunosuppression through HIF-1*α* Signaling Pathway in the Late Phase of Sepsis

**DOI:** 10.1155/2020/4235909

**Published:** 2020-02-07

**Authors:** Li-Li Li, Bing Dai, Yu-Han Sun, Ting-Ting Zhang

**Affiliations:** Department of Respiratory and Critical Care Medicine, The First Affiliated Hospital, China Medical University, Shenyang 110001, China

## Abstract

Severe pneumonia with sepsis is characterized by a dysregulated inflammatory response of endotoxin. In our study, we attempted to investigate the roles of the immune guardian cells (monocytes) in the immune-inflammatory response of severe pneumonia-induced sepsis. We performed analysis in the blood samples of human and animals with ELISA, western blot, flow cytometry (FCM) methods, etc. Results showed that the proinflammatory status shifted to hypoinflammatory phases during the sepsis process. In a clinical study, the levels of IL-1*β*, IL-6, TNF-*α*, etc., except for IL-10, were inhibited in the late phase of sepsis, while, in an animal study, the immune suppression status was attenuated with administration of the adenovirus Ade-HIF-1*α*. Conversely, the amount of IL-10 was lower in the adenovirus Ade-HIF-1*α* group compared with the sepsis model group and the Ade-control group. Moreover, in the clinical study, the programmed cell death-ligand 1 (PD-L1) was overexpressed in monocytes in the late phase of sepsis, while the expression of proteins HIF-1*α* and STAT3 was decreased in the late phase of sepsis. However, in the animal study, we found that the HIF-1*α* factor facilitated the inflammatory response. The expression of the proteins HIF-1*α* and STAT3 was increased, and the PD-L1 protein was decreased with the adenovirus Ade-HIF-1*α* administration compared with the rats without Ade-HIF-1*α* injection and with the Ade-control injection. Additionally, the proteins HIF-1*α* and STAT3 were coregulated at transcriptional levels during the inflammatory responses of sepsis. Taken together, monocytes undergo reprogramming to generate immunosuppression through the HIF-1*α* signaling pathway in the late phase of sepsis.

## 1. Introduction

Severe pneumonia with sepsis caused the highest mortality in intensive care units worldwide due to endogenous endotoxin. Data revealed that there are nearly 5.3 million deaths from severe pneumonia-induced sepsis every year [1]. Sepsis contained two stages: hyperinflammatory and hypoinflammatory phases. During the hyperinflammatory stage, the immune cells are triggered, such as the immune guardian cells: monocytes and neutrophils, which in turn release abundant inflammatory cytokines (IL-1*β*, IL-6, and TNF-*α*), chemokines (CXCL1), myeloperoxidases, and proteases to induce subsequent adaptive immune responses and induce the activated neutrophil degranulation [2, 3]. In the case of the hypoinflammatory phase, the immune system is dysregulated likely owing to the impairment of the phagocytic capacity of monocytes and monocyte-derived macrophages [4]. Studies showed that the expression of major histocompatibility class II antigens and the complement receptor-1 (CR1 and CD35) was reduced [5, 6] in monocytes, the production of inflammatory cytokines TNF-*α* and IL-12 was decreased and the release of anti-inflammatory cytokines IL-10 and PGE2 was enhanced, etc. [7, 8], all of which induced the proliferation and function of T cells and natural killer (NK) cells in the adaptive response [9]. However, some researchers had identified that the predominant immunosuppressed characteristic was that the monocytes cocommunicate with T cells in the sepsis process [10]. The monocytes, a kind of antigen-presenting cells (APC), acted as instigators of T cell suppression in adaptive response by mediating the expression of inhibitory coreceptors such as programmed cell death protein 1 (PD-1) and cytotoxic T lymphocyte-associated protein 4 (CTLA4) [10, 11]. The researches of Avendano-Ortiz et al. [12] and Shalova et al. [13] showed that hypoxia-inducible factor-1*α* (HIF-1*α*) regulated functional reprogramming of monocytes in sepsis to suppress T cells with inhibitory coreceptors, cytokines, and chemokines. It was also shown by Tsukamoto et al. [14] that the myeloid-derived suppressor cells (MDSCs) were more suppressive in nature by upregulating the expression of PD-L1 to impair antigen-specific T cell priming and IgG production in sepsis. Hence, based on bioinformatic analysis of the GSE46955 data [13], we conducted this study to investigate the inflammatory response characteristics of the functional disability in monocytes through the transcription activator hypoxia-inducible factor-1*α* (HIF-1*α*) in mediating the protein signal transducer and activator of transcription 3 (STAT3) during the severe pneumonia with sepsis.

## 2. Materials and Methods

### 2.1. Data Collection

Data extraction of GSE46955 was obtained from the Gene Expression Omnibus (GEO; http://www.ncbi.nlm.nih.gov/geo/) database. From the data, peripheral blood samples were collected from gram-negative sepsis patients during sepsis (sepsis) and following their recovery (recovery) status as well as the healthy donor (control). The platform used in the GSE46955 data was the GPL6104 Illumina HumanRef-8 v2.0 expression beadchip.

### 2.2. Human Blood Samples

Peripheral blood samples of 30 patients diagnosed with severe pneumonia with sepsis were collected in the early phases and late phase. Then, the samples in each phase were equally divided into two groups. 30 blood samples of the healthy participants for the control group were also collected. Besides, the diagnostic criteria of early phase sepsis were patients with symptoms of chills, high fever, poor appetite, fatigue, limb joint soreness, etc., while the patients showed symptoms of respiratory failure, delirium, etc., in the late sepsis phase. Additionally, permission to carry out this study was obtained from the Ethical Committee of our Hospital, and informed consent was obtained from all the patients participating in the study.

### 2.3. Animal Sepsis Model

Eighty SD rats aged 8-10 weeks were purchased from the SiBeiFu Laboratory (Animal Technology Company, China) and equally divided into four groups. The rats in one group were used as the control and the rats in the other three groups were infected with *S. pneumoniae* for more than fifteen days until they had been diagnosed to have pneumonia-induced sepsis. The diagnostic criteria of early sepsis were rats with chills and shortness of breath, and the rats showed coma and were slightly breathless in the late phase of sepsis. Besides, one group of rats from the three experimental groups in the late phase of sepsis were tail vein-injected with the adenovirus Ade-HIF-1*α* (175 *μ*L, at a concentration of 1.5 × 10^6^ PFU) for 5 days of treatment; also, the other sepsis model rats were injected with the same amount of adenovirus control plasmid for 5 days (Gemma Biotechnology Co., Ltd., Shanghai). Thereafter, collection of blood was done from the eyeball and tail vein in the SD rats in the late phase of sepsis and normal control group. Six lung tissues from each group were also obtained.

### 2.4. ELISA Assay

The blood samples obtained both from the patients and the animals were centrifugated at 1500 g for 10 min, two times. Then, the serum of the blood samples was dividedly analyzed with ELISA assay kits as per the manufacturer's protocol to quantify the concentration of IL-1*β* (catalog:70-EK101B-96, homo; catalog:70-EK201B/3-96, mus; MultiSciences, China), IL-18 (catalog: 70-EK118-48, homo; catalog: 70-EK218-96, mus; MultiSciences, China), IL-6 (catalog: 70-EK106/2-96, homo; catalog: 70-EK206/3-96, mus; MultiSciences, China), IL-10 (catalog: 70-EK110/2-96, homo; catalog: 70-EK210/3-96, mus; MultiSciences, China), TNF-*α* (catalog: 70-EK182-96, homo; catalog: 70-EK282/3-96, mus; MultiSciences, China), CCL3 (catalog: 70-EK161-96, homo; catalog: 70-EK261/2-96, mus; MultiSciences, China), and CCL5 (catalog: 70-EK1129-96, homo; catalog: 70-EK2129/2-96, mus; MultiSciences, China) in triplicates.

### 2.5. Western Blot

Lymphocytes were obtained from the blood samples of the patients and the animals with a Ficoll-Hypaque Solution of humans (catalog: p8900, Solarbio, China) and rats (catalog: P8620, Solarbio, China), then the monocytes were isolated from the lymphocytes with the Dynabeads® FlowComp™ Human CD14 kit (catalog: 11367D, Invitrogen, supplementary file ([Supplementary-material supplementary-material-1])). Later, they were lysed in 1 mL of RIPA buffer (Beyotime) for a duration of 20 minutes on ice; besides, the PMSF, a protease inhibitor cocktail, was also mixed in the RIPA buffer. Then, the extracted protein was quantified with a BCA kit (Pierce, Rockford, IL). Afterwards, separation of proteins was carried out with sodium dodecyl sulfate polyacrylamide gel electrophoresis (SDS-PAGE), then the protein gel was transferred to a PVDF membrane (Bio-Rad, Hercules, CA). After that, the protein membranes were blocked with 5% nonfat milk for 1 hour, then the protein membranes underwent incubation overnight at 4°C with primary antibody STAT3 (1 : 500, catalog: 4904S, CST), HIF-1*α* (1 : 500, catalog: 36169S, CST), PD-L1 (1 : 500, catalog: 13684S-homo/29122S-mus, CST), and GAPDH (1 : 1000, catalog: 5174S, CST). Thereafter, the membranes were washed for 3 times in TBST/1% Tween-20, then they were incubated with rabbit polyclonal antibody at 4°C for 60 min. Besides, GAPDH was used as a control. Finally, an ECL detection instrument (Thermo Fisher Scientific) plus a chemiluminescent substrate were added to visualize the immunoreactive bands with Bio-RAP.

### 2.6. Flow Cytometry (FCM)

The monocytes were obtained from peripheral blood samples of the patients with the Ficoll-Hypaque Solution of human (catalog: p8900, Solarbio, China) and the Dynabeads® FlowComp™ Human CD14 kit (catalog: 11367D, Invitrogen); the details of the method were listed in the supplementary file. Then, the obtained monocytes were placed in RPMI-1640 medium with FBS (Thermo Fisher Scientific, USA). Afterwards, the expression of CD274 was analyzed by FCM. The brief methods are listed: the monocytes were fixed and labeled with FITC-conjugated anti-human CD274 antibody (catalog: MA5-16848, Invitrogen), anti-mouse CD274 antibody (catalog: 558065, BD Pharmingen™), and allophycocyanin- (APC-) conjugated anti-human CD14 antibody (catalog: 17-0149-42, Invitrogen). Thereafter, the cells were washed twice and resuspended in PBS for flow cytometry analysis. Finally, the expression of PD-L1 on the monocytes was analyzed with the FACSCalibur (Bioscience, BD, USA) instrument.

### 2.7. Immunofluorescence (IF)

Monocytes were obtained from the patients' blood samples in the primary sepsis stage, late sepsis stage, and healthy control. Then, the cells were fixed with 4% polyformaldehyde for 30 min and permeabilized with 0.1% TritonX-100 for 3 min; afterwards, they were blocked with 10% BSA for 60 min at room temperature. Finally, the cells were incubated overnight with PD-L1 (catalog:13684S, CST) and HIF-1*α* (catalog: 36169S, CST) at 4°C. Besides, the nucleus DNA was stained with DAPI for 5 min (catalog: D9542, Sigma, USA). The images were then visualized under a confocal microscope (Leica scanning microscope, Germany).

### 2.8. Coimmunoprecipitation (Co-IP) Assay

The proteins were extracted from the collected blood samples of the patients with a protein extraction RIPA buffer with PMSF (Beyotime, China). The protein A/G Sepharose (Santa Cruz Biotechnology) was preincubated with anti-HIF-1*α* (catalog: 36169S, CST) or anti-STAT3 (catalog: 4904S, CST) antibody for 60 min at 4°C and IgG antibody as a control. Then, we suspended the bead-antibody complexes with protein lysate and washed the beads with an extraction buffer 3 times. The samples were centrifuged at 3000 g to obtain the immuneprecipitates. Then, the immuneprecipitates were analyzed with the western blot.

### 2.9. Statistical Analysis

The results were analyzed in the one-way ANOVA method with SPSS 19.0 statistical software. The results were considered statistically significant at *P* < 0.05. The differences presented in graphs were analyzed with GraphPad Prism 6.0 software.

## 3. Results

### 3.1. Bioinformatic Analysis of the Data GSE46955

**Figure 1:**
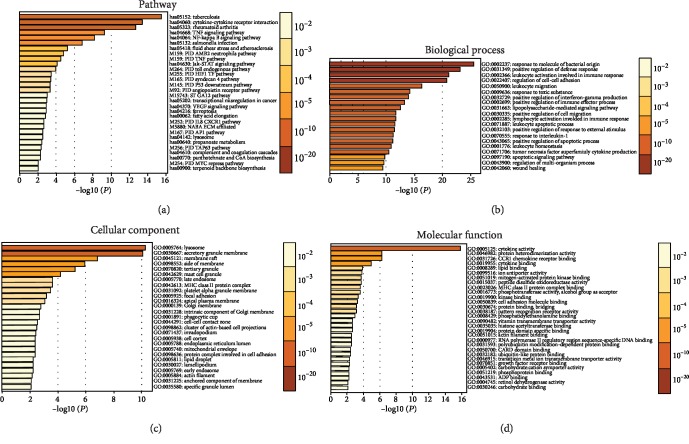
Gene Set Enrichment Analysis of the dataset GSE46955 from the GEO database with Geo2R and DAVID software tools. KEGG and reactome pathway analysis of the major signaling pathways (a). Gene ontology analysis of the differentially expressed genes, the major biological process (b), the major cellular component (c), and the major molecular function (d).

Gene Set Enrichment Analysis of the mRNA expression profiles was carried out for the differentially expressed genes (*P* < 0.05) in monocytes of the gram-negative bacteria-induced sepsis patients from the GEO database (GSE46955). Data showed that the major signaling pathways were the interaction of the inflammatory cytokines, TNF signaling pathway, JAK-STAT signaling pathway, etc. (Figure 1(a)), while the Gene Ontology of the biological processes was mainly a response to bacterial, positive regulation of the defense response, leukocyte activation in immune response, etc. (Figure 1(b)); the cellular components were mainly involved in the cell or granule membrane, etc. (Figure 1(c)); besides, the molecular functions were mainly about cytokine or chemokine activity, etc. (Figure 1(d)).

### 3.2. Immunosuppression of Monocytes via HIF-1*α* Signaling Pathway

**Figure 2:**
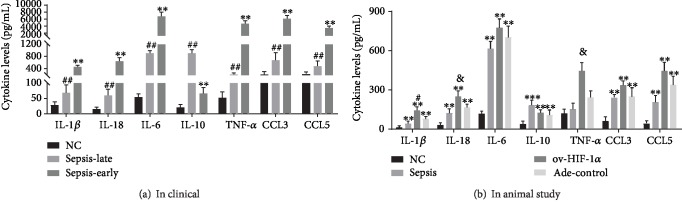
Its cytokine production in the blood serum. (a) In the clinical study, the higher levels of TNF-*α*, IL-1*β*, IL-6, IL-18, CCL3, and CCL5 except for IL-10 in the early phase of sepsis compared with the late phase of sepsis and the healthy participants, *n* = 30. ^∗∗^*P* < 0.01 represented the significant difference; ^##^*P* < 0.01: compared with the healthy participants, the difference was of significance. (b) Ade-HIF-1*α* injection increased the production of TNF-*α*, IL-1*β*, IL-6, IL-18, CCL3, and CCL5 except for IL-10 in the late stage of the sepsis rat model. ^∗∗^*P* < 0.01: compared with the normal control group, the difference was of significance; ^#^*P* < 0.05: compared with the sepsis rat model group and the adenovirus control group; ^&^*P* < 0.05: compared with the sepsis rat model group. Besides, data were presented as mean ± standard deviation, and data was repeated in triplicate.

**Figure 3:**
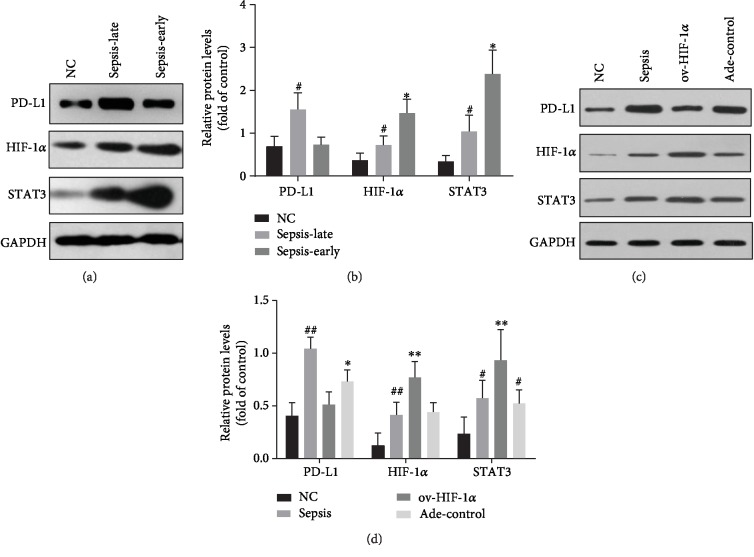
Its expression of proteins in the monocytes. (a) The protein bands of HIF-1*α*, STAT3, and PD-L1 in the clinical study. (b) The downregulated expression of proteins HIF-1*α* and STAT3 in the late phase of sepsis compared with the primary phase of sepsis; ^#^*P* < 0.05 represented significant difference. While the upregulation of the immune checkpoint protein PD-L1 expressed on the monocytes in the late phase of sepsis, ^#^*P* < 0.05 represented significant difference vs. the primary phase of sepsis. ^∗^*P* < 0.05 of proteins HIF-1*α* and STAT3 in the primary phase of sepsis versus the normal control group. (c) The protein bands of HIF-1*α*, STAT3, and PD-L1 in the sepsis rat model study. (d) Upregulated expression of proteins HIF-1*α* and STAT3 and downregulated protein PD-L1 expression in the sepsis rat model with Ade-HIF-1*α* injection; besides the highly expressed protein PD-L1 in the sepsis rats model, ^##^*P* < 0.05 of PD-L1 and HIF-1*α* versus the normal control group and ov-HIF-1*α* adenovirus-injected group, ^∗^*P* < 0.05 of PD-L1 versus the normal control group; ^∗∗^*P* < 0.05 of HIF-1*α* and STAT3 versus the normal control group, sepsis rat model group, and adenovirus control group; ^#^*P* < 0.05 of STAT3 versus the normal control group and ov-HIF-1*α* adenovirus-injected group; data were presented as mean ± standard deviation, and analysis was replicated in triplicate.

**Figure 4:**
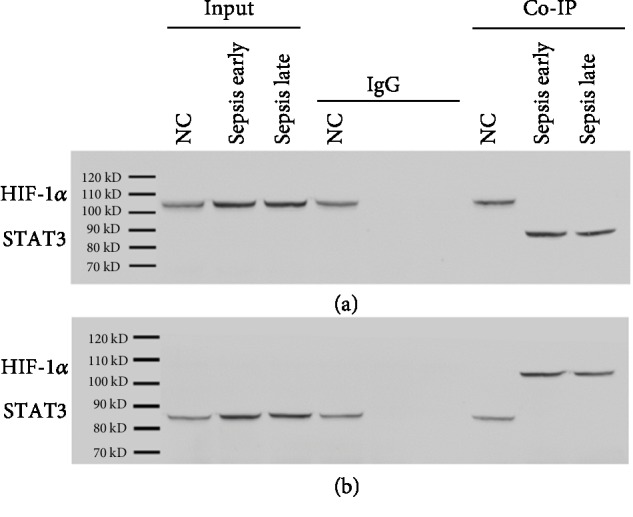
Coimmunoprecipitation assay of the proteins HIF-1*α* and STAT3. (a, b) In clinical blood samples, the proteins HIF-1*α* and STAT3 were coregulated at transcriptional levels.

The analysis of inflammatory cytokine generation from blood samples of patients and the normal control showed that during the early phases of sepsis, there was a proinflammatory status with higher levels of TNF-*α*, IL-1*β*, IL-6, IL-18, CCL3, and CCL5, compared with the late phase of sepsis except for the production of IL-10 (Figure 2(a)), while, in the animal study, the administration of adenovirus Ade-HIF-1*α* alleviated the immune suppression status in the late stage of sepsis and results showed that TNF-*α*, IL-1*β*, IL-6, IL-18, CCL3, and CCL5 levels were higher in the Ade-HIF-1*α* group than in the Ade-control group, the sepsis model group without adenovirus injection, and the normal control group (Figure 2(b)), while the amount of IL-10 was increased more obviously in the sepsis rat model group compared with the Ade-control group (Figure 2(b)). Additionally, in the clinical study, the western blot analysis of the proteins HIF-1*α* and STAT3 showed that the expression of protein STAT3 was significantly reduced in the late phase of sepsis compared with the primary phase (Figures 3(a) and 3(b)). Meanwhile, in the animal study, data presented that administration of Ade-HIF-1*α* resulted in an increased expression of HIF-1*α* and STAT3 compared with the sepsis rat model group and the Ade-control group (Figures 3(c) and 3(d)). Conversely, in the clinical study, the expression of protein PD-L1 was upregulated in the late phase of sepsis, while, in the animals' study, the protein PD-L1 expression was higher in the sepsis rat model group compared with the Ade-control group and the Ade-HIF-1*α* group (Figure 3). Interestingly, with the coimmunoprecipitation assay, results showed that proteins HIF-1*α* and STAT3 coregulated at the transcriptional level in the immune process of sepsis (Figures 4(a) and 4(b)). Therefore, monocytes undergo immunosuppression in the sepsis late phase through the HIF-1*α* signaling pathway.

### 3.3. Overexpression of Immune Checkpoint Protein PD-L1

**Figure 5:**
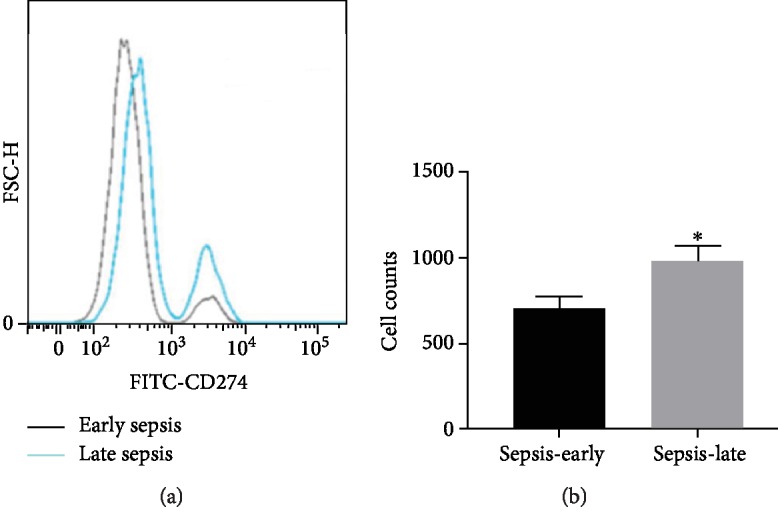
(a) Flow cytometry analyzed the expression of PD-L1 in the blood samples of sepsis patients (*n* = 30); the dark line represented the early phase of sepsis, while the blue line represented the late phase of sepsis. (b) ^∗^*P* < 0.05 stands for the significant difference.

**Figure 6:**
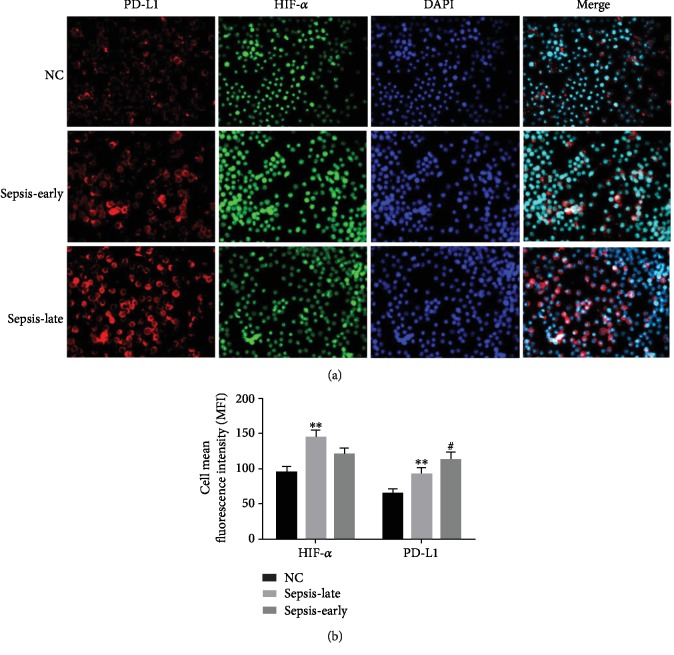
Its immunofluorescence analysis of proteins HIF-1*α* and PD-L1 (200x) in the clinical study. (a) The protein HIF-1*α* (red) is located in the nuclear, the immune checkpoint protein PD-L1 (green) is expressed on the monocyte membrane, and DAPI was used to stain the nucleus (blue). (b) Reducing the mean fluorescence intensity (MFI) of protein HIF-1*α* in the late phase of sepsis, ^∗∗^*P* < 0.05 of HIF-1*α* and PD-L1 versus the normal control group represented significant difference, while increasing the fluorescence intensity (MFI) of protein PD-L1 in the late phase of sepsis versus the early phase, ^#^*P* < 0.05 of PD-L1 versus normal control group represented significant difference.

**Figure 7:**
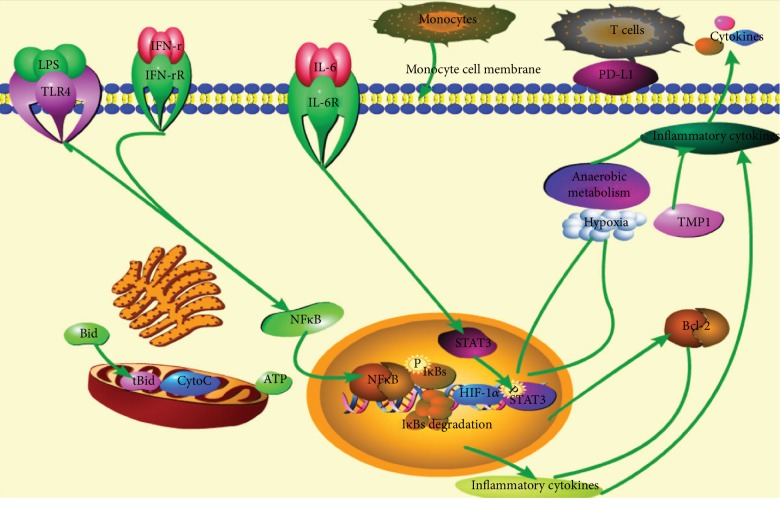
The inflammatory response through the HIF-1*α* signaling pathway. The inflammatory cytokine IL-6 and other inflammatory signals bind with the receptors expressed on the immune cells (monocytes, etc.) to activate the downstream signals. In our study, the proteins HIF-1*α* and STAT3 were activated to induce inflammatory response in the early phase of sepsis. While, in the late phase of sepsis, the immune checkpoint protein PD-L1 expressed on the monocyte membrane was upregulated to generate immunosuppression.

As we had found that the monocytes undergo immunosuppression through the HIF-1*α* signaling pathway (Figure 3), the further study of FCM analysis identified that PD-L1 expressed on the monocytes was higher in the late phase of sepsis compared with the early phase (Figure 5). Additionally, the immunofluorescence results displayed that the protein HIF-1*α* expressed on the nuclear and PD-L1 expressed on the monocyte membrane (Figure 6). Therefore, monocytes undergo immunosuppression in the late phase of sepsis through the HIF-1*α* signaling pathway, which induced the upregulation of protein PD-L1 expressed on the monocytes (Figure 7).

## 4. Discussion

Based on this study, we further confirmed that sepsis was an uncontrolled inflammatory response syndrome. During the sepsis process progression, it shifted to the immunosuppression status. Results showed that the primary phase of sepsis was associated with hyperinflammation with higher amounts of proinflammatory cytokine secretion. Conversely, there was an increased production of anti-inflammatory cytokine IL-10 in the late phase of sepsis. This conclusion is in consistent with the finding of Shalova et al. [13]. Moreover, we also found that the HIF-1*α* was activated in hypoxic conditions during the primary phase in sepsis, which then coactivated the signal transducer and activator of transcription 3 (STAT3) to engage in the inflammatory process. HIF-1 protein is a heterodimer consisting of two subunits: HIF-a and HIF-1b. Under hypoxia condition, the nonhydroxylated HIF-1*α* is not degraded. Then, the unmodified protein dimerizes with HIF-1b and p300 (or CBP) binding to HIF-1*α* to activate the transcription of HIF-1 target genes in its downstream [15, 16]. In a tumor study, researchers Niu et al. showed that STAT3 activation was proposed to be upstream of HIF-1*α* and regulated HIF-1*α* transcription in its pathway [17]. Hence, in sepsis, STAT3 also mediated HIF-1*α* transcriptional activity in its signaling pathway. Furthermore, in our clinical study, results also showed that the proinflammatory response was suppressed in the late phase of sepsis. Data revealed it not only inhibiting the proinflammatory cytokine/chemokine production, but also upregulating the expression of protein PD-L1. However, in the late stage sepsis rat model study, results identified that hypoxia-inducible factor-1*α* adenovirus injection alleviated the immunosuppression status along with higher secretion of proinflammatory cytokines/chemokines and lower expression of protein PD-L1. Hence, the immune checkpoint protein PD-L1 expressed on the monocyte membrane also participated in the sepsis immunosuppression response. Dissatisfied, in our study, we did not identify which gene or protein directly regulated the expression of PD-L1 in the HIF-1*α* downstream signaling pathway.

Immune checkpoints (ICs) played pivotal roles in immune surveillance in cancer [18]. They were ligands of lymphocyte receptors engaging in modulating the duration and initiation of adaptive immune response [19]. Therefore, it is possible that the induction of immune tolerance in sepsis inhibited the T cell effector response through PD-L1 in monocytes, which was in line with the work of Chang et al. [20] and Zhang et al. [21]. It was declared that PD-L1 was a sepsis phenotype playing roles in the communication among monocytes and T lymphocytes. Therefore, further studies are required to reveal the mystery of PD-L1 in the communication among monocytes and T lymphocytes. In addition, a study by Deng et al. [22] claimed that the core circadian clock gene, BMAL1, upregulates the pyruvate kinase M2 (PKM2) to prevent the development of sepsis by coregulating programmed cell death-ligand 1 (PD-L1) in macrophages. Hence, this is also a new approach for us to direct our further study.

The inflammatory response through the HIF-1*α* signaling pathway is mainly due to IL-6/STAT3 axis and NF-*κ*B. Studies indicated that the axis NF-*κ*B2/p100 [23] or the phosphorylated NF-*κ*B [24, 25] and the NF-*κ*Bp65/RelB heterodimers [26] played pivotal roles in endotoxin tolerance during sepsis in human monocytes. Furthermore, the study of Qin et al., clearly declared that SIRT5 competed with SIRT2 to interact with NF-*κ*Bp65 to increase acetylation of p65, which promoted the production of cytokines against the progression of sepsis [27]. Hence, it is possible that the protein NF-*κ*B also engages in the sepsis immune response in the HIF-1*α* signaling pathway, which also awaits further study. Taken together, based on our study, the immune checkpoint protein PD-L1 overexpressed in monocytes during the late phase of sepsis contributed to immunosuppression in severe pneumonia-induced sepsis through the HIF-1*α* signaling pathway.

## 5. Conclusion

Monocytes undergo immunosuppression in the late phase of sepsis through the HIF-1*α* signaling pathway, which thus inhibited the production of proinflammatory cytokines and induced the upregulation of protein PD-L1 expressed on the monocytes.

## Data Availability

The data used to support the findings of this study are all included within the article.

## Supplementary Materials

Supplementary Materials The protocols of isolating monocytes.
