# Supplementary material for: Monocytes Undergo Functional Reprogramming to Generate Immunosuppression through HIF-1α Signaling Pathway in the Late Phase of Sepsis
Source: Mediators Inflamm. 2020 Feb 7;2020:4235909. doi: 10.1155/2020/4235909 (PMC7029303; doi:10.1155/2020/4235909)
Supplement: Supplementary Materials — The protocols of isolating monocytes. [file 4235909.f1.doc]

**The protocols of isolating monocytes**

**Wash the beads**

1. Resuspend the beads in the vial (vortex >30 sec or tilt and rotate for 5 min).

2. Transfer (187.5uL/5 mL of human) or (150uL/4mL of mice) volume of beads to a tube.

3. Add the volume of Isolation Buffer (1 mL) and resuspend the beads.

4. Place the tube in a magnet for 1 min and discard the supernatant.

5. Remove the tube from the magnet and resuspend the washed beads in the isolation Buffer (1mL) .

**Prepare cells**

• keep all buffers and reagents cold (2°C to 8°C) during the entire isolation process.

• Collect whole blood sample into a blood collection tube containing an appropriate

anticoagulant, heparin.

• Prepare 5mL (human) or 4 mL (mice) whole blood samples in each tube.

• Wash the whole blood samples once prior to use with Isolation Buffer (PBS) to remove soluble CD14 in the sample, and centrifuge 350 × g for 10 min at 2°C to 8°C without brakes. Remove pellet back to the original starting volume (leave approximately 1 cm above the red blood pellet).

**Isolate cells from whole blood**

1. Transfer 5 mL pre-cooled and preferable washed whole blood to a tube on ice and

add 12.5 μL FlowComp™ Human CD14 Antibody.

2. Mix well and incubate for 10 min at 2°C to 8°C.

3. Fill up the tube with Isolation Buffer and mix well, followed by centrifugation for

15 min at 350 × g at 2°C to 8°C with no brakes.

4. Aspirate the supernatant and discard the volume added in step 3 (but keep at

least 1 cm above cell pellet to avoid monocyte loss).

5. Add 187.5 μL resuspended FlowComp™ Dynabeads® and mix well by vortexing.

6. Incubate for 15 min at 2°C to 8°C under rolling and tilting.

7. Add 10 mL Isolation Buffer, mix gently and place the tube in the magnet for

minimum 3 min.

8. While the tube is still in the magnet, carefully remove and discard the supernatant containing the CD14 negative cells. Be careful not to disturb the bead pellet on the tube wall. Use a thin pipette.

9. Repeat steps 7-8 twice to wash the bead-bound CD14+ cells a total of three times.

Use only half the amount of Isolation Buffer (5 mL) in the last wash. These steps are critical to obtain a high purity of isolated cells.

**Release cells from whole blood**

10. Resuspend in 2.5 mL FlowComp™ Release Buffer and pipet 3–4 times.

11. Incubate for 10 min at 2°C to 8°C under rolling and tilting.

12. Pipet 10 times to efficiently release the cells and place in a magnet for 1 min.

Avoid foaming.

13. Transfer the supernatant containing the bead-free CD14+ cells to a new tube and

again place on the magnet for 1 min to remove any residual beads. Transfer again

the supernatant containing the bead-free cells to a new tube.

14. Add 5mL Isolation Buffer followed by centrifugation for 8 min at 350 × g.

Discard the supernatant and resuspend the cell pellet in preferred cell medium.

Keep the monocytes on 2°C to 8°C for FCM analysis.
